# Altered Humoral Immune Responses and IgG Subtypes in NOX2-Deficient Mice and Patients: A Key Role for NOX2 in Antigen-Presenting Cells

**DOI:** 10.3389/fimmu.2018.01555

**Published:** 2018-07-11

**Authors:** Julien Cachat, Christine Deffert, Marco Alessandrini, Pascale Roux-Lombard, Audrey Le Gouellec, Marie-José Stasia, Stéphanie Hugues, Karl-Heinz Krause

**Affiliations:** ^1^Department of Pathology and Immunology, Geneva University Hospitals (HUG) and Faculty of Medicine, University of Geneva, Geneva, Switzerland; ^2^Division of Laboratory Medicine, Department of Genetic and Laboratory Medicine and Department of Medical Specialities, Geneva University Hospitals (HUG) and Faculty of Medicine, University of Geneva, Geneva, Switzerland; ^3^TheREx (Thérapeutique Recombinante Expérimentale), Laboratoire TIMC-IMAG, University Grenoble Alpes, CNRS, Grenoble, France; ^4^Laboratoire BEP, Pôle Biologie, CHU Grenoble Alpes, Grenoble, France

**Keywords:** NOX2, B cells, T cells, immunoglobulin, antigen-presenting cells, curdlan, IgG subtype

## Abstract

Chronic granulomatous disease (CGD) is a primary immunodeficiency resulting from loss of function mutations in the reactive oxygen species generating phagocyte NADPH oxidase (NOX2). CGD patients are prone to infection, but also have an increased susceptibility to autoimmune diseases. The aim of this study was to investigate the role of NOX2 in the regulation of specific immunity. In both CGD patients and NOX2-deficient mice, we observed an alteration in the basal proportions of IgG subtypes. Upon immunization with curdlan—a dectin 1 agonist—NOX2-deficient mice showed increased production of IgG2c compared to controls, and restimulation of lymph node-derived cells led to increased production of IFNγ, but not IL-5, indicative hallmark of an enhanced Th1 response. T cell activation was increased in NOX2-deficient mice and a similar trend was observed *in vitro* when T cells were co-cultured with NOX2-deficient bone marrow-derived cells. In contrast, no difference in T cell activation was observed when NOX2-deficient T cells were co-cultured with wild-type BMDC. Following stimulation of NOX2-deficient dendritic cells (DCs), no difference in costimulatory molecules was observed, while there was an increase in the release of Th1-driving cytokines. In summary, both CGD patients and CGD mice have an altered IgG subtype distribution, which is associated with an increased IFNγ production. Thus, NOX2 within DCs appears to be an important regulator at the interface of innate and specific immunity, especially after activation of the dectin 1 pathway, limiting immune activation and the development of autoimmunity.

## Introduction

Chronic granulomatous disease (CGD) is a primary immunodeficiency disorder leading to life-threatening bacterial and fungal infections. The genetic cause of CGD is the loss of function mutations in genes coding for the phagocyte NADPH oxidase NOX2. NOX2 is a multi-subunit enzyme generating reactive oxygen species (ROS) and is an essential component of the neutrophil oxidative burst leading to microbial killing (1). While the role of NOX2-derived ROS in innate neutrophil activity is well established, NOX2 has other important functions in immune system regulation. Early reports of CGD had described an increase in immunoglobulin levels (2), but this feat has since received little attention. B cells express a functional NOX2 (3). NOX2 may have a role in antibody production, but only a few studies reported an impact of NOX2 deficiency on B cell biology and immunoglobulin production. CGD mice show increased immunoglobulin responses to injection of collagen (4) and to UV-inactivated bacteria (5). One study in NOX2-deficient mice observed an enhanced antibody production in response to T cell-independent antigens (6). To date, no study has carefully analyzed the effect of NOX2 on IgG subclass production.

In addition, recent observations argue in favor of an excessive inflammatory response and autoimmunity in CGD (7). CGD patient show increased prevalence of autoimmune diseases, including systemic lupus erythematosus, juvenile rheumatoid arthritis, and idiopathic thrombocytopenic purpura (8–11). Autoantibodies play a role in the pathogenesis of these immune diseases (12). The sera of patients with CGD also show high levels of anti-*Saccharomyces cerevisiae*, anti-OmpC, or anti-CBir1 antibodies, which are associated with Crohn disease (13). In rodents, a seminal paper by the team of Rikard Holmdahl demonstrated that a loss of function polymorphism in the *Ncf1* gene—which codes for the p47^phox^ subunit of NOX2—is a main driver of experimental rheumatoid arthritis (14, 15). Since then, observation converges toward a role of NOX2-derived ROS in T cell activation. Indeed adoptive T cell transfer from arthritic NOX2-deficient mice is sufficient to induce the disease in healthy wild-type (WT) mice (14). Thus, NOX2-derived ROS limit T cell activation, although the underlying mechanisms are still incompletely understood. NOX2-derived ROS, generated either by T cells themselves or antigen-presenting cells (APCs), might directly inhibit T cells, possibly through surface oxidation (16), ROS inhibition of lymphocyte ion channels (17), or other redox-sensitive signaling elements (18). Alternatively, NOX2-derived ROS might play a role in APCs and indirectly affect T cell function. For example, a recent study reported altered antigen processing, resulting in a different epitope repertoire in NOX2-deficient dendritic cells (DCs) (19), while another study has shown that oxidative modification of presented autoantigens enhances T cell response (20). NOX2-derived ROS appear to fundamentally control specific immune responses as mice deficient in *Ncf1* also exhibit an increased sensitivity to autoimmune encephalitis (EAE) (21) and NOX2-deficient mice an increased sensitivity to lupus erythematous (22). Interestingly, a recent human genetic study also found that a missense variant in *NCF1* is associated with susceptibility to multiple autoimmune diseases (23). Altogether, these studies suggest that the link between NOX2 and autoimmune disease is not limited to CGD patients, but also exists for less severe polymorphisms of the NOX2 system. Nevertheless, although patients with NOX2 deficiency present with increased risk to infection due to the impaired neutrophil oxidative burst, autoimmune features are not always visible and probably require specific additional stimuli. We have previously shown that dectin-1 activation strongly induces a CGD-associated hyperinflammation. Injection of curdlan, a β-glucan, which is a potent activator of dectin-1, results in a massive subcutaneous swelling and high levels of IL-6 and IFNγ in NOX2-deficient mice, while lipopolysaccharide was inactive (24). Altogether, the current literature indicates clinical and experimental links between NOX2-dependent ROS generation, production of immunoglobulins, specific hyperinflammatory states, and the development of autoimmune diseases.

In the present study, we measured IgG subclasses in the sera of NOX2-deficient mice and in CGD patients and detected altered IgG subtype production in NOX2 deficiency. We also addressed experimentally the activation of T cells following immunization with an ovalbumin-derived peptide (OVA_323–339_) and the impact of specific adjuvants *in vivo* and in BMDC and T cell co-culture experiments. Our results point toward a key role of dectin-1-dependent NOX2 in DCs in limiting T cell activation, IFNγ release, and the production of Th1-driving cytokines. This suggests that NOX2-deficient DCs release increased amount of Th1-driving cytokines, leading to the release of an increased amount of IFNγ, which in turn may drive a higher IgG2c generation by B cells.

## Materials and Methods

### Mice

C57Bl/6j (WT), B6.129S-Cybbtm1Din/J (NOX2KO), and B6.Cg-Tg(TcraTcrb)425Cbn/J (OTII) were purchased from The Jackson Laboratory and bred at the Animal Production facilities of the University of Geneva. Double OTII/NOX2KO-mutant mice were obtained by breeding B6.129S-Cybbtm1Din/J mice with B6.Cg-Tg(TcraTcrb)425Cbn/J mice. For the experiments, mice of age 8–12 months were used. The protocol was approved by the office cantonal vétérinaire du Canton de Genève, Switzerland (authorization no. 23624).

### Patients

Patients were diagnosed as having CGD on the basis of their clinical symptoms and the inability of their phagocytes to generate ROS detectable by the dihydrorhodamine (DHR) flow cytometric test and the nitroblue tetrazolium dye reduction slide test. Blood samples were obtained from the CGD patients with appropriate institutional informed consent. Peripheral blood samples taken from healthy donors were obtained from the "Etablissement Français du sang" at the Grenoble University Hospital, France after their informed consent.

### Flow Cytometry

Cells were suspended at 10^6^/ml in FACS buffer (PBS with 0.5% bovine serum albumin (BSA) and 5 mM ethylenediaminetetraacetate (EDTA)). Fc receptors were blocked by a 10 min incubation at 4°C with the mouse BD Fc block (BD Biosciences, USA) at a dilution of 1:100. The cells were then washed with FACS buffer and centrifuged at 5,000 rpm for 5 min. Cells were then resuspended in FACS buffer with the antibody of interest and incubated for 15 min at 4°C. After incubation, the cells were washed with FACS buffer, centrifuged at 5,000 rpm for 5 min and resuspended in FACS buffer for flow cytometry analysis.

#### Immunization

Wild-type and NOX2KO mice were immunized by subcutaneous injection into the outer ear, using 50 µl of 1 µg ovalbumin protein and curdlan (Sigma-Aldrich, USA) (100 µg/ml) or Alum (ThermoFisher Scientific, USA) (50%) as adjuvant, which was diluted in PBS. After 10 and 14 days, blood was collected from the caudal vein. Serum was obtained by coagulation and centrifugation for 2 min at 2,500 rpm, and stocked at −20°C until ELISA was performed. Serum from non-immunized mice was also collected to determine basal levels of the IgG subgroup.

### Hyperinflammation Measurements

Ear thickness was measured using a caliper before immunization (basal ear thickness), at day 10 and 14 after immunization. The change in ear thickness (Δ ear thickness) was obtained by subtracting the basal ear thickness to the values obtained on days 10 and 14.

### ELISA

High binding ELISA 96-well microplate (Corning^®^, Sigma-Aldrich, USA) were coated overnight at 4°C with either 10 µg/ml of ovalbumin (Sigma-Aldrich, USA) diluted in PBS to measure ovalbumin-specific antibody, or with purified goat anti-mouse total IgG (Biolegend, USA, dilution 1:100,000) to measure basal level of IgG subgroup. Plates were washed three times with PBS containing 0.05% tween-20 to remove unbound ovalbumin. A volume of 100 µl of PBS-BSA 1% was used for blocking for 1 h at room temperature. Fifty microliters per well of diluted serum sample were added to corresponding wells in duplicate. A pool of serum was used to obtain a standard curve by serial dilution. Sample dilutions were chosen to obtain a signal within the linear phase of the standard curve. The plates were incubated at room temperature for 1 h and washed three times. To measure ovalbumin-specific antibody, anti-mouse IgG2c, anti-mouse IgG2b, and anti-mouse IgG3 (Biolegend, USA) or anti-mouse IgG1 (ThermoFisher Scientific, USA) coupled to peroxidase was added to wells at the dilution of 1:1,000 for anti-mouse IgG2c and IgG1 and 1:500 for anti-mouse IgG2b and IgG3. Those secondary antibodies were incubated for 1 h at room temperature. For the measure of basal level of IgG subgroups, anti-mouse IgG2c (Biolegend, USA) or anti-mouse IgG1 (ThermoFisher Scientific, USA) or anti-mouse IgG3 (ThermoFisher Scientific, USA) or anti-mouse IgG2b (ThermoFisher Scientific, USA) coupled to peroxidase was added to wells at a dilution of 1:1,000 and incubated for 1 h at room temperature. Plates were washed three times and 50 µl of streptavidin coupled with horse-peroxidase was added to the plates, and incubated at room temperature for 20 min. After three washes, the signal was revealed by adding 50 µl of tetramethylbenzidine (TMB) and the plates were incubated at room temperature for 15 min in the dark. The reaction was stopped by adding 25 µl of H_2_SO_4_ 2N. Optical density at 492 nm was measured using the fluoSTAR OPTIMA (BMG Labtech, Germany) plate reader.

### Measurements of Serum Immunoglobulin G and A and IgG Subclass Levels

Sera from normal (*n* = 6) and CGD patients (*n* = 16) were collected and stored at −80°C before testing. Levels of serum immunoglobulin G and A were measured on a Dimension VISTA^®^ system (Siemens, Germany) with IGG Flex^®^ and IGA Flex^®^ reagent cartridges according to the dimension VISTA^®^ Operator’s Guide. Serum IgG subclasses were measured by nephelometric assay with human IgG subclass liquid reagent kits (Siemens, Germany, including N Latex IgG1, N Latex IgG2, N Latex IgG3, and N Latex IgG4) on the automatic protein analyzer BN Prospect-II (Siemens, Germany) according to the manufacturer’s instructions.

### Anti-Nuclear Antibody (ANA) and Anti-Neutrophil Cytoplasmic Antibodies (ANCA) Determination

Anti-nuclear antibody was determined by indirect immunofluorescence (IFI) on Hep2 slides and ANCA by IFI on ethanol- and formalin-fixed neutrophils (Inova Diagnostics, USA). Briefly, for both types of autoantibodies, the corresponding slides were incubated with serial dilutions of serum in phosphate buffered saline with 10% Tween for 30 min, washed with PBS-Tween, and incubated with fluorescein-isothiocyanate (FITC)-conjugated goat polyclonal anti-human IgG containing Evans blue (Inova Diagnostics, USA). After washing steps, slides were mounted with glycerine mounting medium and cover slips and read using a fluorescence microscope Axioscope 2plus (Carl Zeiss Microscopy GmBh, Germany). Sera were considered positive for autoantibodies when a staining was observed for a dilution equal to or greater than 1/80 for ANA and 1/40 for ANCA.

### Lymph Node Cellularity

Immunized mice were sacrificed 14 days after immunization and draining lymph nodes (cervical lymph node) and non-draining lymph nodes (inguinal lymph node) were dissected. Lymph nodes were digested with 1 mg/ml collagenase D (ThermoFisher Scientific, USA) and 10 µg/ml DNAse I (ThermoFisher Scientific, USA) in 2 ml HBSS at 37°C for 40 min. Digestion was stopped by adding 15 ml of a PBS-BSA 1% plus 5 mM EDTA solution and digested lymph nodes were passed through a 70 µm cell strainer. The total number of cells obtained after lymph node digestion and cell subgroup were analyzed by flow cytometry with the following antibodies: anti-CD4 Percp (1:200), anti-CD8 PE (1:100), and anti-IgD fitc (1:100) (ThermoFisher Scientific, USA).

### Lymph Node Cell Restimulation

Lymph node cells were plated in a 96-well plate at a concentration of 2 × 10^6^ cells per well and restimulated with 100 µg/ml of ovalbumin. 3 days after restimulation, supernatants were analyzed for the presence of IFNγ and IL5 using commercially available ELISA kits (ThermoFisher Scientific, USA), according to the manufacturer’s instructions.

### Bone Marrow-Derived DCs (BMDCs)

Hind leg bones of WT and NOX2KO mice were dissected and the bone marrow flushed with RPMI medium, complemented with 1% streptomycin–penicillin and 10% fetal bovine serum, and plated in a petri dish. Bone marrow cells were centrifuged at 5,000 rpm for 5 min and the pellet resuspended in a solution of 155 mM NH_4_Cl and 12 mM NaHCO_3_ for 1 min to lyse red blood cells. 20 ng/ml of GM-CSF (Peprotech) was added to the medium to drive differentiation to DCs. Medium and GM-CSF were renewed at day 3 and 6. At day 10, purity of BMDCs was verified by flow cytometry using anti-CD11c APC (1:100) antibody.

### Isolation of OTII and OTII/NOX2 T Cells

Lymph node of OTII or OTII/NOX2 mice were dissected and passed through a 15 µm cell strainer to obtain a single cell suspension of lymph node cells. T cells were isolated using a microbead-based T cell isolation kit (Miltenyi Biotec, Germany), according to the manufacturer’s instruction. Purity of the isolated T cells was verified by flow cytometry using anti-CD4 PE antibody (1:200).

### *In Vitro* Co-Culture Experiments

Wild-type and NOX2KO BMDCs were plated in RPMI (completed with 10% fetal bovine serum, 1% streptomycin–penicillin, 50 µM β-mercaptoethanol, and 1 mM sodium pyruvate) in a 96-well plate at a concentration of 10^4^ cells per well. Curdlan at 5 µg/ml was added to the wells for BMDC activation. Finally, increasing concentrations (5–500 nM) of OVA_323–339_ peptide were added to the culture. After 1 day, BMDCs were harvested and washed twice with the prepared RPMI medium to remove the curdlan and peptide. Then, freshly isolated OTII or OTII/NOX2 T cells were added to the wells with the same medium preparation at a concentration of 10^5^ T cells per well. For the experiment on T cell proliferation, freshly isolated T cells were first labeled with 5 µM carboxyfluorescein succinimidyl ester (CFSE) for 10 min at 37°C and washed three times. T cell activation was assessed by flow cytometry after 16 h of co-culture and after staining with anti-CD4 PercP (1:200) and anti-CD69 PE (1:200) antibody. T cell proliferation was assessed by flow cytometry after 4 days of co-culture and after staining with anti-CD4 PercP. The proliferation index and percent of dividing cells were calculated with FlowJo Software.

### *In Vivo* T Cell Activation

Wild-type and NOX2KO mice were immunized with 0.5 µg of OVA_(323–339)_ and 100 µg/ml of curdlan in PBS subcutaneously in the outer ear (50 µl of the solution was injected by ear). 1 day after immunization, freshly isolated OTII or OTII/NOX2KO T cells were labeled with 5 µM CFSE and injected intravenously in immunized mice. For T cell activation, draining lymph nodes were dissected 16 h after T cell adoptive transfer. Lymph nodes were then scratched and lymph node cells were stained with anti-CD4 PercP (1:200) and anti-CD69 PE antibody (1:200) for flow cytometry analysis. For T cell proliferation, lymph node dissection was performed 3 days after immunization and was stained with anti-CD4 PercP antibody. For comparison of T cell activation and proliferation between OTII and OTII/NOX2KO T cells, the experiment were performed similarly except that OTII T cells were labeled with CFSE as described above and OTII/NOX2KO T cells were labeled with 1 µM CellTracker™ Orange Cmtmr ((5-(and-6)-(((4-chloromethyl)benzoyl)amino)tetramethyl-rhodamine)) (ThermoFisher scientific, USA) at 37°C for 15 min, and then washed three times.

### T Cell Activation and Survival in Presence of H_2_O_2_

Freshly isolated T cells were plated in a 96-well plate at a concentration of 10^5^ T cell per well in the same culture medium as for co-culture experiments. Anti-CD3 (0.5 µg/ml) and anti-CD28 (2 µg/ml) antibody were then added to the culture to activate T cells. At the same time, increasing concentrations of H_2_O_2_ from 16 to 150 µM were added. After 16 h of treatment, T cells were stained with anti-CD4 PercP (1:200) antibody and anti-CD69 (1:200) PE antibody, and analyzed *via* flow cytometry. For T cell survival, activated T cell was stained with propidium iodide.

### BMDC Activation

Bone marrow-derived dendritic cells were plated in a 96-well plate in same medium than for co-culture experiments at a concentration of 10^5^ cells per well. Curdlan at concentration of 50, 25, and 5 µg/ml was added to the culture for BMDCs activation. After 24 h, BMDCs were harvested and supernatants were kept for further analysis at −20°C. BMDCs were stained with anti-CD11c Percp (1:200) or anti-CD11c APC (1:100), anti-DC-Sign APC (1:100), anti-ICOSL PE (1:100), anti-PD-L1 PE (1:100), anti-CD80 PE (1:400), anti-CD86 fitc (1:200), and anti-MHCII PE (1:1000). IL1β, IL10, and IL6 were measured by ELISA kit (eBioscience) in the supernatant, according to the manufacturer’s instructions.

### Statistical Analysis

All data were analyzed using GraphPad Prism software. For sample sizes of *n* ≥ 7, data were first tested for normality using the Shapiro–Wilk test. If normally distributed, the Student’s *t*-test was used for statistical comparison. For datasets not normally distributed and where sample sizes *n* ≤ 7, the Mann–Whitney *U* test was used.

## Results

We first analyzed immunoglobulin levels in serum samples from healthy donor and CGD patients. As expected, higher levels of immunoglobulin where found in patients with CGD (Table S1 in Supplementary Material). Six CGD patients had increased IgA serum levels of which four were adults. In contrast, of the six CGD patients that had higher levels of serum IgM, four were below the age of two. For serum IgG, five CGD patients had levels above the normal range, of which three were adults and two children. When investigating the presence of autoantibodies, 6 out of 16 CGD samples (37%) were positive for ANCA, while 3 out of 16 samples (19%) were positive for ANA (Table S1 in Supplementary Material). Thus, we confirmed previous observations that CGD patients are prone to hypergammaglobulinemia and a higher frequency of autoantibodies.

**Figure 1:**
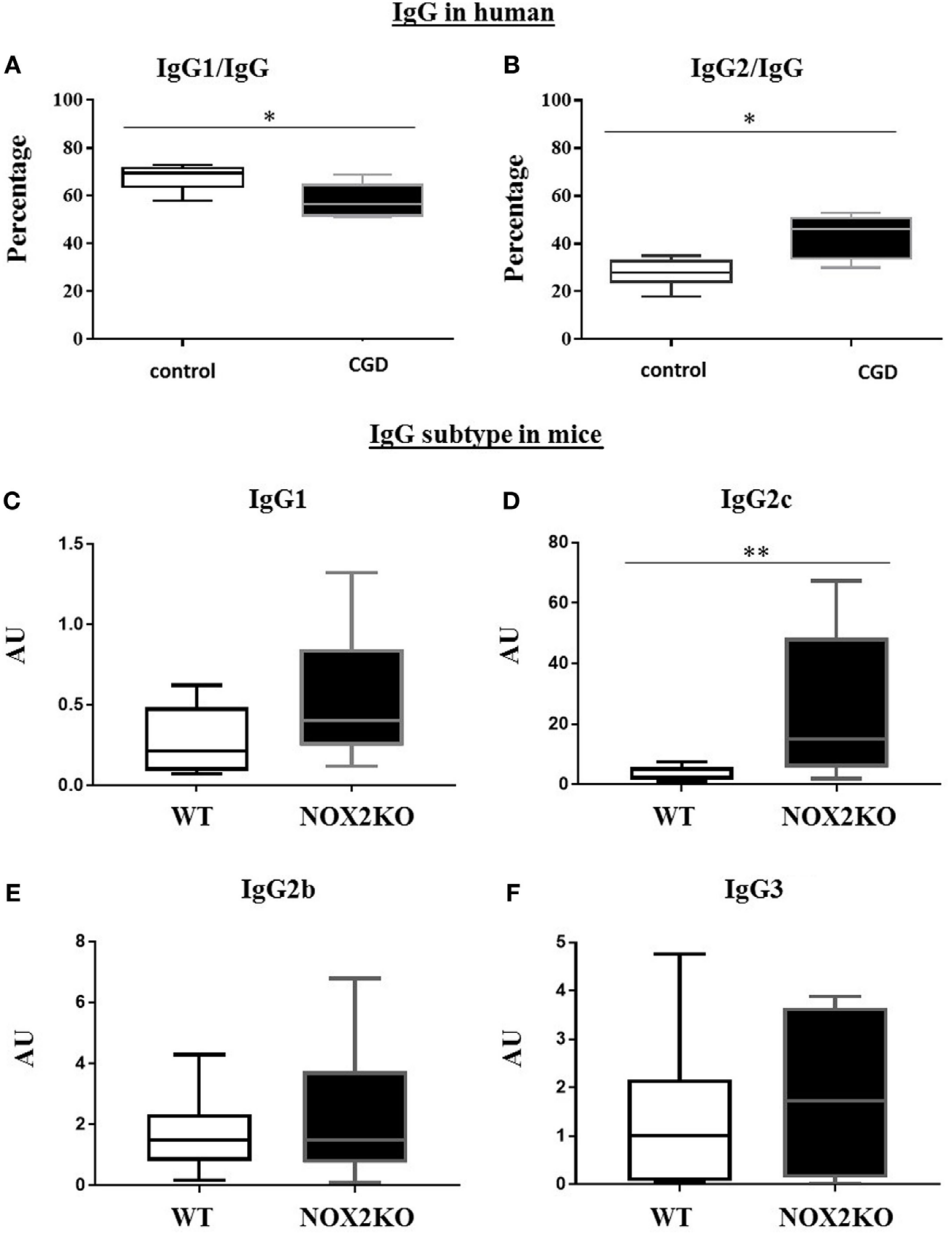
Relative levels of IgG1 and IgG2 in CGD patients and healthy donors and basal levels of immunoglobulin subtypes in wild-type (WT) and NOX2-deficient mice. **(A,B)** Proportion of serum IgG1 and IgG2 subtypes relative to total IgG. Basal level of IgG1 **(C)**, IgG2c **(D)**, IgG2b **(E)** and IgG3 **(F)** in WT and NOX2 deficient mice (NOX2KO). *p < 0.05 and **p < 0.01.

We further characterized different IgG subtypes and compared the relative amounts of IgG1 and IgG2. Since no reference values for the ratios of IgG1/IgG and IgG2/IgG exist, we compared these values for CGD patients relative to a control group of comparable ages. In the control group, IgG1 represented ~70% of total IgG. In CGD patients, these levels were statistically significantly decreased (*p* = 0.026; Figure 1A). In contrast, IgG2 represented ~30% of total IgG in controls, and were significantly increased to ~40% in CGD patients (*p* = 0.01; Figure 1B).

To determine whether the differences in IgG subtypes were also present in NOX2-deficient mice, we measured the basal levels of each IgG subtype (IgG1, IgG2b, IgG2c, and IgG3) in NOX2-deficient and WT mice (Figures 1C–F). Similar to our human data, levels of some, but not all, IgG subtypes were impacted by the presence of NOX2. We did not find significant differences in IgG1, IgG2b, and IgG3 levels between WT and NOX2-deficient mice (Figures 1C,E,F). However, levels of IgG2c were significantly increased in NOX2-deficient mice (*p* < 0.008; Figure 1D). Thus, an alteration of IgG subtypes was observed in human CGD samples and in NOX2-deficient mice.

**Figure 2:**
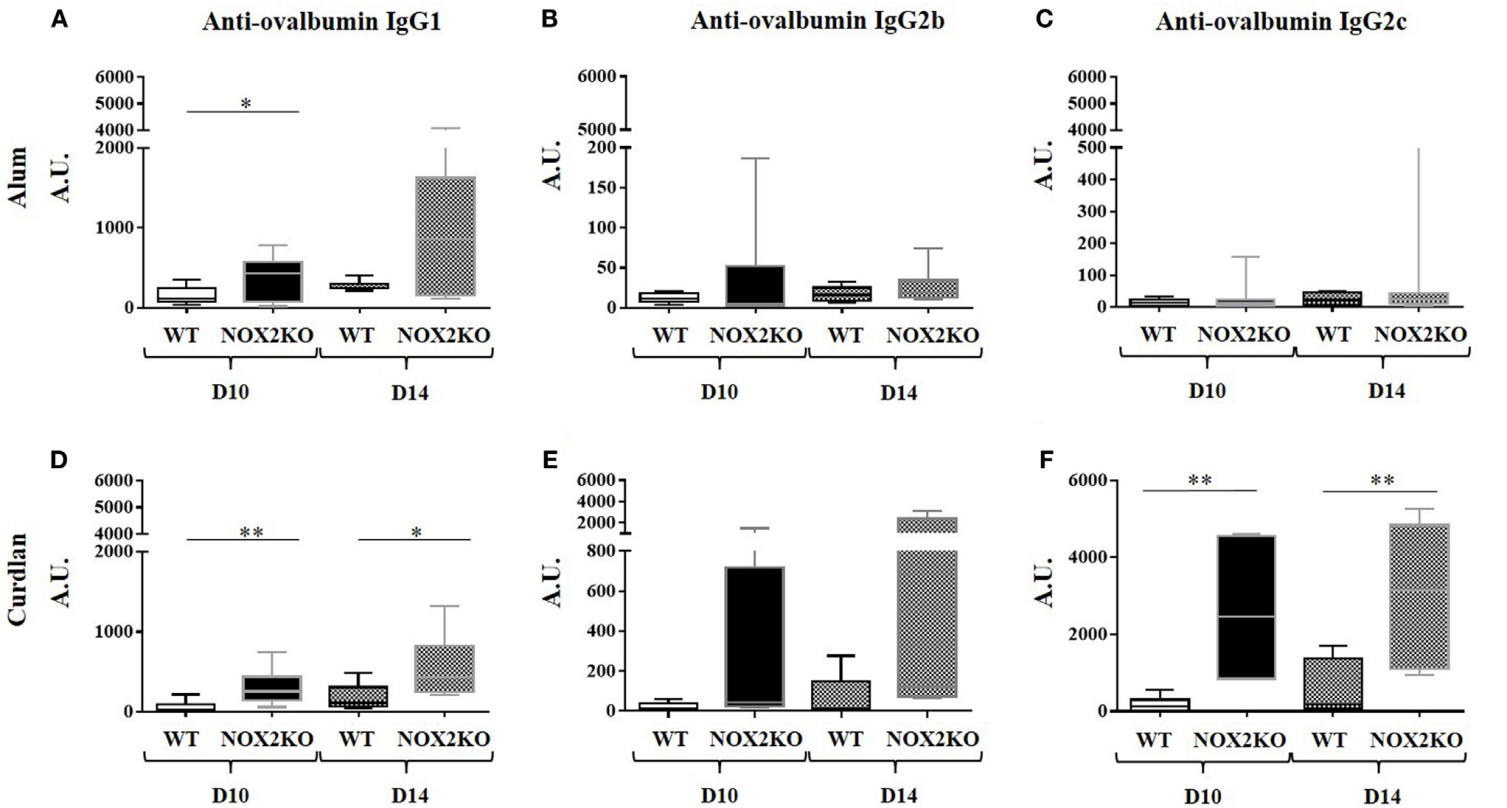
Enhanced immunoglobulin generation in CGD mice: IgG subtype and adjuvant dependence. Mice were immunized by subcutaneous injection into the outer ear of ovalbumin with either alum **(A–C)** or curdlan **(D–F)** as adjuvant. Levels of Anti-ovalbumin IgG1 **(A,D)**, IgG2b **(B,E)** and IgG2c **(C,F)** were measured 10 and 14 days following immunization (*n* = 12). *p < 0.05; **p < 0.01; and ***p < 0.001.

In order to examine how NOX2 deficiency affects the production of antibodies, we compared the levels of antigen-specific IgG in response to immunization of control and NOX2-deficient mice. As a model antigen, we used ovalbumin, and either alum or curdlan as adjuvants. The two different adjuvants were chosen for the following reasons. Alum is a standard adjuvant used in most routine experiments, and it mostly induces IgG1 response. In contrast, curdlan is an adjuvant that privileges IgG2c responses. Immunization was performed by injection in the ear skin. Different investigations were performed 10 and/or 14 days after immunization, including ear thickness, serum IgG levels, and lymph node analysis (cellularity and cytokines). Anti-ovalbumin IgG subtypes were examined. Prior to immunization, no anti-ovalbumin IgG was detected (data not shown). After immunization, the level of anti-ovalbumin IgG3 was still below detection level (data not shown). In control mice, immunization with either alum or curdlan as adjuvant led to the generation of modest, but clearly detectable, amounts of anti-ovalbumin IgG1. In immunized NOX2-deficient mice, the IgG1 levels were broadly increased in comparison to control mice (Figures 2A,D). The situation of anti-ovalbumin IgG2b was slightly more complex. With alum as adjuvant, the level of anti-ovalbumin IgG2b remained low for all samples (Figure 2B). With curdlan as adjuvant, even though most of the samples had a low level of IgG2b, one sample in WT mice and two samples in NOX2KO mice exerted levels at least 10 times higher (Figure 2E). In contrast, there was a notable impact of NOX2 deficiency on the pattern of IgG2c production, which was primarily influenced by the choice of adjuvant. With alum as adjuvant, IgG2c responses were completely absent in both WT and NOX2-deficient mice (Figure 2C). However, with curdlan as adjuvant, while only minor elevated IgG2c levels were observed in WT mice, considerable and statistically significant IgG2c elevations were detected in the sera of NOX2-deficient mice (Figure 2F).

**Figure 3:**
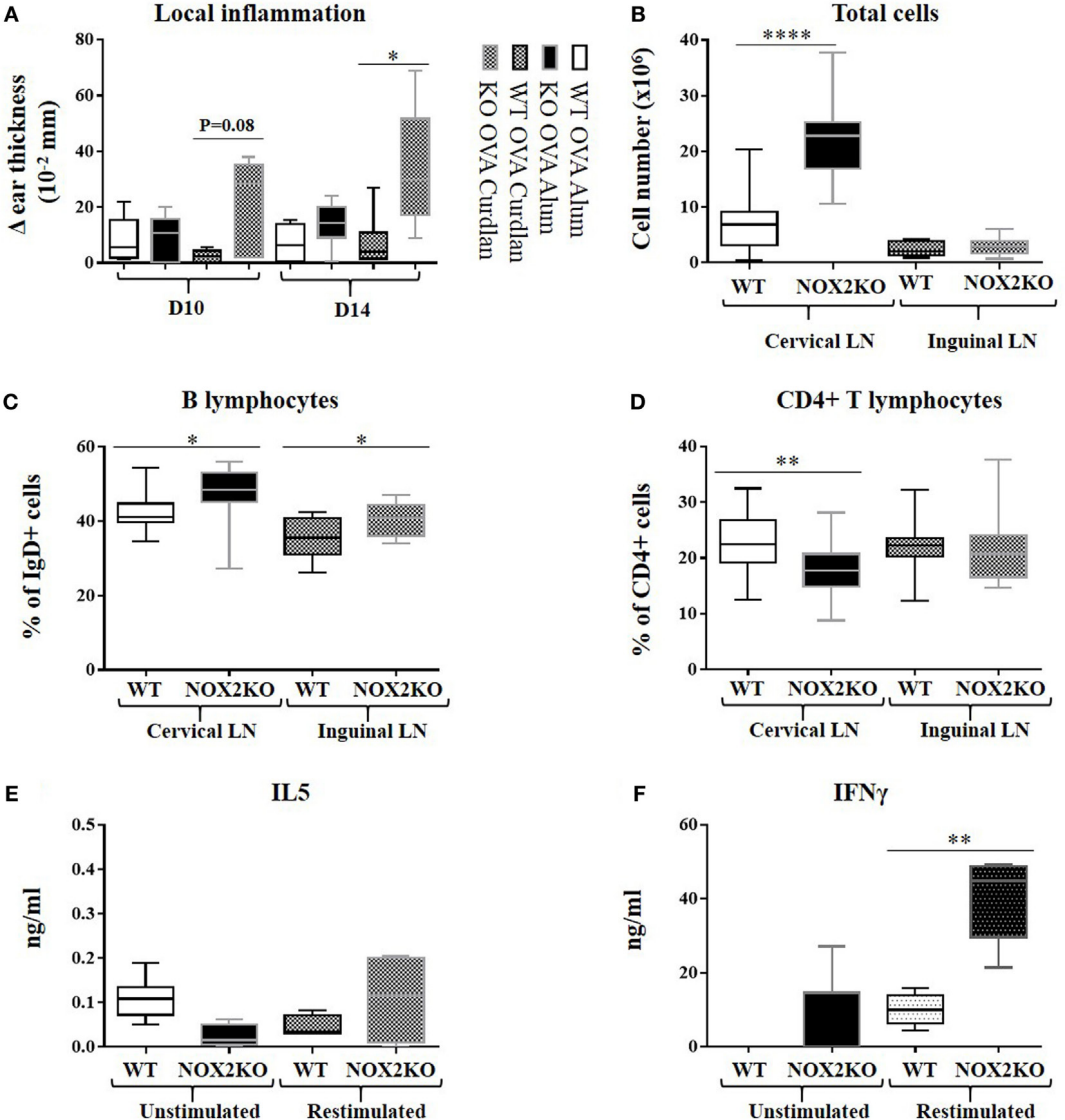
Enhanced inflammation and release of inflammatory mediators upon immunization of CGD mice. Mice were immunized by subcutaneous injection into the outer ear of ovalbumin with either alum or curdlan as adjuvant. **(A)** Ear thickness, reflecting the inflammatory response, was measured 10 and 14 days following immunization. **(B–D)** Draining cervical and non-draining inguinal lymph nodes were removed 14 days post immunization, dissociated, and analyzed by flow cytometry: **(B)** total cell number; **(C)** percentage of IgD-positive B cells; **(D)** percentage of CD4-positive T cells (*n* = 10). **(E,F)** Draining lymph nodes were isolated 10 days post immunization with ovalbumin and curdlan, and release of IL5 **(E)** and IFNγ **(F)** was measured 24 h after restimulation of lymph node homogenates with ovalbumin (*n* = 10). *p < 0.05; **p < 0.01; and ****p < 0.0001. The legend next to panel **(A)** refers only to this panel.

The mechanisms leading to antibody production are complex and involve different cell types to reach a full humoral response. In order to address the mechanisms behind the marked increased IgG2c production in NOX2-deficient mice, we investigated inflammation resulting from the immunization, both locally and in draining lymph nodes. Measurement of ear thickness was performed as a read-out for the local inflammatory reaction. Ear thickness, prior to immunization, did not differ between WT and NOX2-deficient mice (data not shown). After intra-auricular immunization, a moderate increase in ear thickness was measured in WT mice (0.1–0.2 mm), which was similar when both alum and curdlan adjuvants were used. In NOX2-deficient mice, the ear thickness was strongly increased with curdlan as adjuvant, but not with alum (Figure 3A), indicating that curdlan induces an increased inflammatory reaction upon immunization.

To characterize the inflammatory response, we investigated the total cell numbers of draining (cervical) and non-draining (inguinal) lymph nodes after immunization with ovalbumin and curdlan. In draining lymph nodes, there was a threefold median increase in cellularity (*p* < 0.0001) in NOX2-deficient mice when compared to WT mice (Figure 3B). In non-draining lymph nodes, the total number of cells was relatively low and there was no difference between WT and NOX2-deficient mice. We next analyzed the different subpopulations of lymph node cells. The proportion of CD4 positive T cells in draining lymph nodes of NOX2-deficient mice was significantly lower (*p* = 0.001) when compared to control WT mice (Figure 3D), whereas the proportion of IgD-positive B cell population was significantly higher (Figure 3C). Eventhough the proportion of CD4-positive T cells is decreased in NOX2-deficient immunized mice, it is important to notice that the absolute numbers of both CD4-positive T cells and IgD-positive B cells are increased in NOX2-deficient mice compared to WT (Figure S1 in Supplementary Material). Taken together, our data demonstrate that immunization of NOX2-deficient mice with curdlan as adjuvant leads to an increase of the total cell number in the draining lymph nodes, which is predominantly accounted for by an increase in IgD-positive B cells.

We next investigated the levels of cytokine release by cells isolated from draining lymph nodes, as an indirect measure of T cell differentiation and/or proliferation in response to immunization. For these experiments, mice were immunized with ovalbumin and curdlan, as described above. 10 days post immunization, draining lymph nodes were mechanically dissociated and cultured for 72 h in the presence or absence of ovalbumin. Low-level IL-5 production persisted for all conditions (Figure 3E), suggesting limited Th2 response. However, significant differences were observed with respect to the IFNγ levels (Th1): restimulation with ovalbumin led to a moderate increase in IFNγ levels from WT lymph node cells, while the production of IFNγ was strongly increased in NOX2-deficient lymph node cells (Figure 3F). These results suggest that immunization of NOX2-deficient mice with curdlan as adjuvant preferentially leads to an antigen-specific Th1 response.

**Figure 4:**
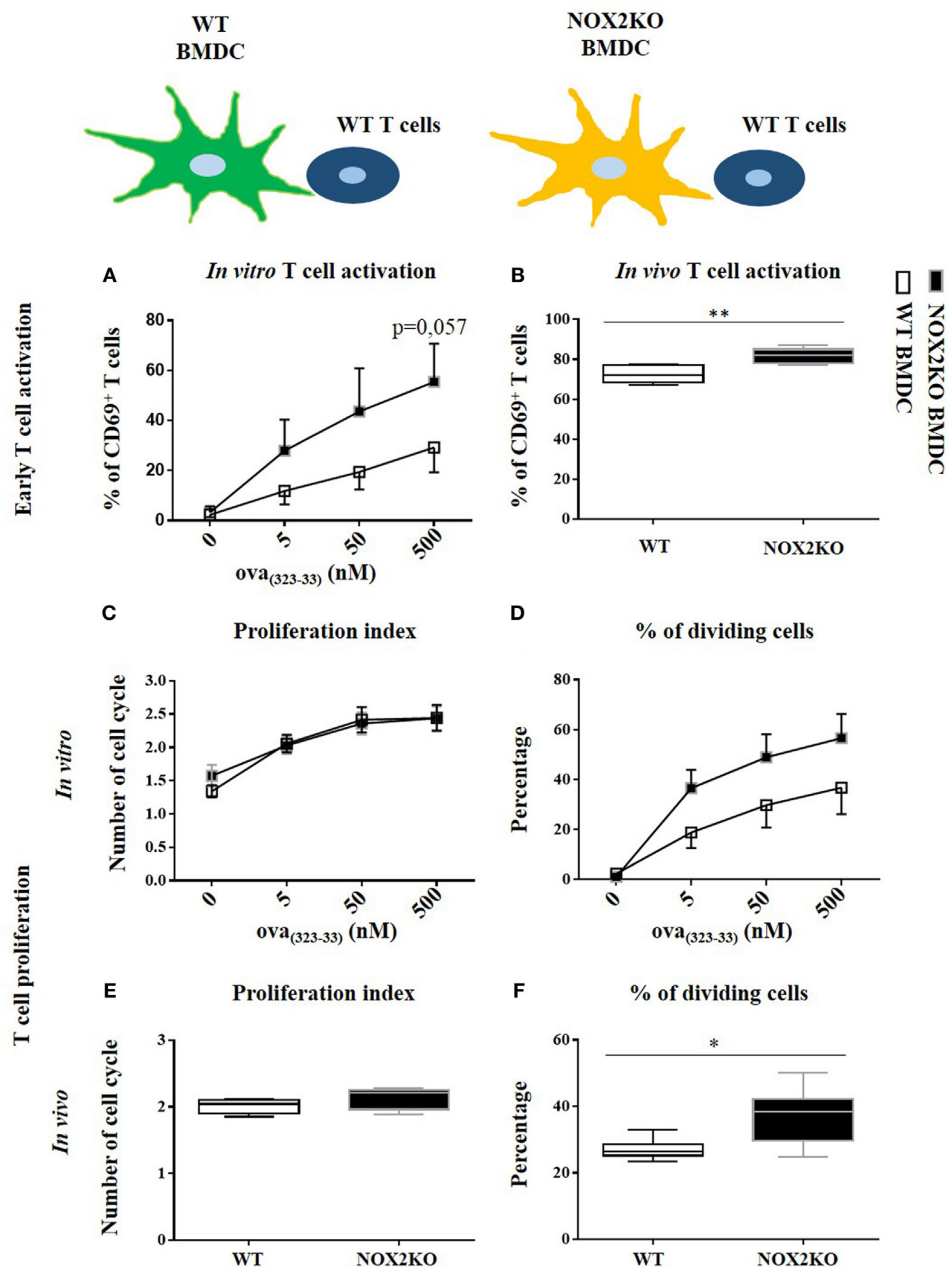
Effect of NOX2 in dendritic cells on T cell activation and proliferation *in vitro* and *in vivo*. OTII T cells were co-cultured with wild-type (WT) or NOX2KO bone marrow-derived dendritic cells (BMDCs) in the presence of different concentrations of OVA_(323–339)_ peptide (5, 50, and 500 nM) for *in vitro* experiments **(A,C,D)**, or were labeled with carboxyfluorescein succinimidyl ester (CFSE) and injected in OVA_(323–339)_ pre-immunized WT or NOX2KO mice for *in vivo* experiments **(B,E,F)**. T cell activation was determined 16 h after co-culture or 16 h after T cell injection by measuring the upregulation of CD69 by flow cytometry. **(A)** Percentage of CD69^hi^CD4^hi^ T cells *in vitro*. **(B)** Percentage of CD69^hi^CD4^hi^CFSE^hi^ T cells *in vivo*. For T cell proliferation, T cells were labeled with CFSE for the *in vivo* and *in vitro* experiments. The proliferation index and the percent of dividing cells were determined by the dilution of the CFSE signal in the OTII T cell population: **(C,E)** proliferation index and **(D,F)** percent of dividing cells *in vitro* **(C,D)** (*n* = 7) and *in vivo* **(E,F)**. *p < 0.05 and **p < 0.01.

To perform a more in-depth analysis of T cell responses, we used an *in vitro* co-culture system with OVA_(323–339)_-specific T cells (OTII T cells) and WT or NOX2-deficient (NOX2KO) BMDCs. T cell activation was assessed by the upregulation of CD69 at the surface of CD4 T cells after 16 h of co-culture. CD69 is an early T cell activation marker. In absence of OVA_(323–339)_ peptide, barely no CD69^hi^ CD4^+^ T cells were detectable (Figure S2 in Supplementary Material). Addition of different OVA_(323–339)_ concentrations led to low to moderate T cell activation (identified as CD69^hi^ CD4^+^ T cells) in a dose-dependent manner (Figure S2 in Supplementary Material). For each OVA_(323–339)_ peptide concentration tested, there was a trend toward a higher percentage of CD69^hi^ T cells when T cells were co-cultured with NOX2KO BMDCs compared to WT BMDCs. This difference was highest at 500 nM of OVA_(323–339)_ peptide, where a 2.5-fold higher T cell activation was observed for NOX2KO BMDCs compared to WT DCs (Figure 4A).

To further characterize the effect of NOX2 in BMDCs on T cell responses, we measured T cell proliferation. For that purpose, we used the property of the CFSE vital dye, for which fluorescence is equally divided by two in daughter cells after each cell division, allowing to study cell division. In the absence of antigen, T cells were not activated and did not enter into cell division. There was, therefore, only one peak of CFSE fluorescence (Figure S3 in Supplementary Material). With the addition of OVA_(323–339)_ peptide to the co-culture, T cells started to divide and different peaks of CFSE fluorescence were detected. The percentage of cells that entered into division increased as a function of OVA_(323–339)_ peptide concentration (Figure S3 in Supplementary Material). Co-culture of T cells with either WT or NOX2KO BMDCs did not change their proliferation index (Figure 4C). In contrast, there was also a trend toward a higher percentage of T cells that entered into cell cycle when T cells were incubated with NOX2KO BMDCs compared to WT BMDCs (Figure 4D). Therefore, the absence of NOX2 in BMDCs might facilitate the entry of T cells into cell division but once T cells went into cell division, they behaved similarly when incubated with either WT or NOX2KO DCs. These results confirm the above observations, and suggest an increase in early T cell activation with NOX2KO DCs compared to WT DCs.

Given the observed trends in our *in vitro* data, we wanted to independently confirm our results and further address the question *in vivo*. WT or NOX2KO mice were immunized subcutaneously in the outer ear with curdlan and OVA_(323–339)_ peptide. 1 day later, CFSE-labeled OTII T cells were injected intravenously. The T cell response was analyzed in the draining and the non-draining lymph nodes at day 1 for early T cell activation (CD69 expression) and at day 3 for T cell proliferation (CFSE dilution). The upregulation of CD69 on CFSE^hi^ CD4^+^ T cells in the draining lymph nodes demonstrated an activation of T cells 1 day after immunization, which was not the case in non-draining lymph nodes (data not shown). Recapitulating our *in vitro* co-culture assays, the percent of CD69^hi^ CD4^+^ CFSE^hi^ T cell was higher 1 day after immunization of NOX2KO mice compared to WT controls (Figure 4B). At day 3, the percentage of CFSE-labeled OTII T cells that had initiated proliferation was markedly higher in CGD mice, as compared to WT (Figure 4F), whereas there was no difference in the proliferation index (Figure 4E). Thus, both *in vitro* and *in vivo*, the number of CD4^+^ T cells upregulating the early activation marker and entering into cell division is increased when the APCs do not express NOX2.

**Figure 5:**
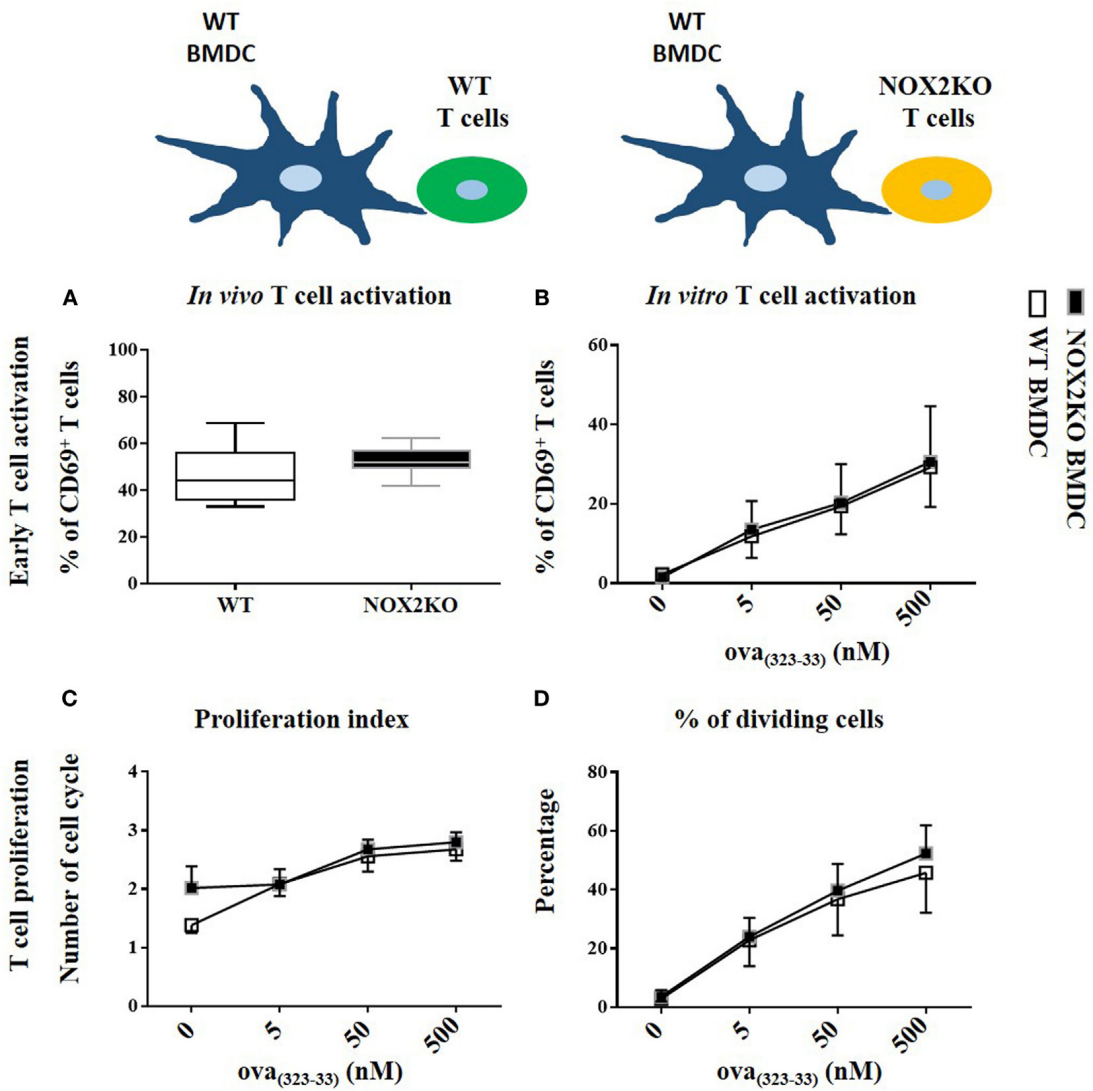
NOX2 in T cells does not impact T cell activation and proliferation *in vitro* and *in vivo*. Percent of CD69^hi^ in NOX2 and wild-type CD4^+^ T cells *in vitro* **(A)** and *in vivo* **(B)**. Proliferation index **(C)** and percent of dividing cells **(D)** at the different concentration of OVA_323–339_ peptide *in vitro* (*n* = 7).

To determine whether NOX2 expression in T cells contribute to the above observations, we assessed whether NOX2-deficient and WT T cells behave differently *in vitro* and *in vivo*. For that purpose, we crossed OTII mice with NOX2KO mice, as a source of T cells that were OVA-specific (OTII) and NOX2-deficient. For *in vitro* experiments, OTII WT and OTII NOX2KO T cells were co-cultured with curdlan-activated OVA_(323–339)_ peptide loaded WT BMDCs. For *in vivo* experiments, OTII WT and OTII NOX2KO T cells were labeled with two different fluorescent vital dyes: a mixture of OTII WT and OTII NOX2KO T cells (ratio 1:1) was injected intravenously in WT mice that had been previously immunized with the OVA_(323–339)_ peptide and curldan. Using this system, we were able to analyze and compare the activation of both WT and NOX2KO T cells in the same recipient mice. Both *in vitro* and *in vivo* experiments showed neither difference in T cell activation nor T cell proliferation between WT and NOX2KO CD4^+^ T cells. Percentages of CD69^hi^ CD4^+^ T cells were identical when OTII WT and OTII NOX2KO where used, *in vivo* and *in vitro* (Figures 5A,B), and both the proliferation index and the percent of dividing cells were identical *in vitro* (Figures 5C,D). Thus, T cells from control and NOX2-deficient mice responded similarly to activation by DCs, refuting claims of a cell autonomous effect of NOX2 in T cells.

**Figure 6:**
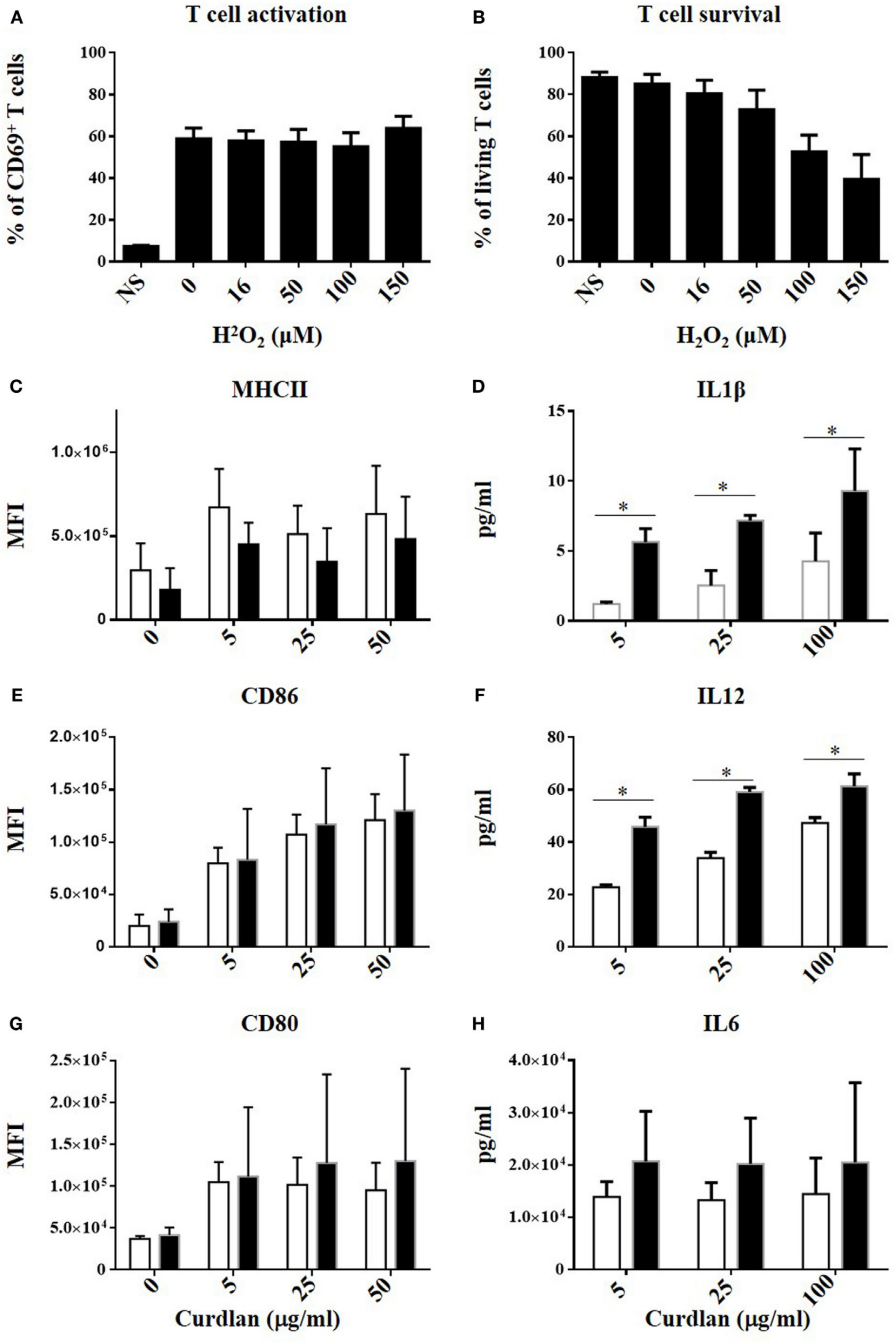
No direct effect of H_2_O_2_ on T cell activation but increased IL12 and IL1β production by NOX2KO bone marrow-derived dendritic cells (BMDCs) after activation by curdlan. Effect of increasing concentration of H_2_O_2_ on CD69 upregulation **(A)** and survival **(B)** in anti-CD3/anti-CD28 activated CD4^+^ T cell (*n* = 4). Expression level of MHCII **(C)**, CD86 **(E)**, and CD80 **(G)** in curdlan-activated wild-type (WT) and NOX2 BMDC was analyzed by flow cytometry (*n* = 4; MFI, mean fluorescent intensity). Level of IL-1β **(D)**, IL-12 **(F)**, and IL-6 **(H)** present in the supernatant of curdlan-activated WT and NOX2KO BMDC (*n* = 3).

Finally, several NOX2-dependent mechanisms limiting T cell activation by BMDCs were tested: (i) a paracrine effect of BMDC-derived H_2_O_2_ on T cells, (ii) a NOX2-dependent modulation of costimulatory molecules in BMDCs, which are known to influence the strength of T cell activation, and (iii) an impact of NOX2 on the release of T cell modulatory cytokines by BMDCs. For the first mechanism, the possible effect of BMDC-derived H_2_O_2_ on T cells was assessed by activating T cells from WT mice with anti-CD3/anti-CD28 antibodies and exposing them to increasing concentrations of exogenous H_2_O_2_. The percentage of CD69^hi^ CD4^+^ T cells was not affected by H_2_O_2_, even at cytotoxic concentrations of H_2_O_2_ (Figures 6A,B)_._ This excludes a direct role of BMDC-derived H_2_O_2_ on T cells. To test the second possibility, co-stimulatory molecule expression at the BMDC surface was analyzed by measuring the level of expression of MHCII, CD80, CD86, ICOSL, PDL1, and DC-SIGN after exposure of BMDCs to different concentrations of curdlan. All these molecules were similarly expressed by NOX2KO and WT BMDCs (Figures 6C,E,G; Figures S4A–C in Supplementary Material), ruling out an effect of NOX2 on the phenotype of BMDCs. To test the third mechanism, the production of T cell modulatory cytokines by NOX2 and WT BMDCs was measured after stimulation with curdlan. Whereas no effect of NOX2 deficiency was observed for the production of Th1 unrelated cytokines (IL-6 and IL-10) (Figure 6H; Figure S4D in Supplementary Material), NOX2-deficient BMDCs produced increased amounts of the pro-Th1 cytokines IL-12 and IL-1β compared to WT BMDCs (Figures 6D,F). In conclusion, our data neither favor a direct impact of NOX2-derived H_2_O_2_ on lymphocytes, nor a signaling through costimulatory receptors. In contrast, our data indicate an important role of NOX2 in BMDCs for limiting the release of pro-Th1 cytokines.

## Discussion

Our study represents, to the best of our knowledge, the first analysis of the impact of the phagocyte NADPH oxidase (NOX2) on the pattern of IgG subtype repartition. The results show that NOX2 not only limits the amount of circulating antibodies, but also contributes to the determination of the composition of IgG subtypes. Indeed, upon immunization, NOX2-deficient mice show increased IgG2c levels. As underlying mechanisms, we propose that the increased release of Th1-driving cytokines from NOX2-deficient APCs leads to enhanced IFNγ production by activated T cells, which may in turn promote IgG2c production by B cells.

First reports describing CGD in the late 1950s had already described increased immunoglobulin levels (2), even before the lack of microbicidal activity of phagocytes in CGD was discovered (25). Subsequently, however, the increased immunoglobulin levels in CGD patients received little attention and were mostly attributed to repeated infections. Our study confirms that even in the absence of an experimental immunization, CGD mice have altered immunoglobulins levels. We found that serum levels of IgG2c were increased in a statistically significant manner. Note that these mice never developed a clinical apparent infection. Thus, we assume that the daily host–pathogen interaction, even in the absence of infection or controlled immunization is sufficient to reveal the tendency of CGD mice to produce a different subset of IgG. However, the controlled immunization performed in this study allowed us to further characterize the immune phenotype. Interestingly, the impact of NOX2 deficiency on IgG subtype production depends on the nature of the adjuvant. Alum (known to drive a Th2 response) promotes a modest IgG1 production in both WT and NOX2-deficient mice. Neither WT nor NOX2-deficient mice produced IgG2c with alum as adjuvant. Curdlan has been described to induce both Th1 and Th17 responses, with the Th1 response being predominant (26). However, in a previous study, our laboratory showed that IFNγ but not IL-17 is increased in NOX2-deficient mice after injection of curdlan in the outer ear of mice (27). Accordingly, we found a low level of the Th2-driven anti-ovalbumin IgG1 subtype in both WT and NOX2-deficient mice. In contrast, curdlan induces a marked increase in the production of IgG2c in WT mice, which was strikingly enhanced in NOX2-deficient mice. Thus, NOX2 deficiency by itself does not alter the type of the immune response elicited by a given adjuvant, but rather amplifies its response. Our results suggest the following scenario: in WT mice, curdlan activation of the dectin-1 pathway in DCs leads to production of Th1-driving cytokines, such as IL-12 and IL-1β, the magnitude of these cytokines being limited by NOX2-dependent ROS generation. In the absence of NOX2, the Th1 response is not kept under control, resulting in an enhancement of this pathway, and a subsequent deviation toward IgG2c responses. It will be of major interest to identify the ROS target in DCs. Typically, ROS signaling may occur through oxidation of redox-sensitive cysteines, for example, within the catalytic site of protein phosphatases (28). The identification of the ROS target would potentially pave the way toward innovative immunomodulatory treatments. Interestingly, a study showed an enhanced IL12 production after stimulation of NOX2-deficient BMDCs with IFNγ/LPS of NOX2 and this effect was correlated with a decreased p38-MAPK (29). Therefore, it would be interesting to investigate this pathway in order to elucidate the mechanism by which NOX2 controls IL12 production.

Understanding which NOX2 expressing cells influence the antibody responses is essential, the more relevant candidates being APCs, T cells, and B cells. Our results argue against a role for NOX2 in T cells. This is supported by previous publications suggesting that NOX2 is virtually not expressed in T cells (30). Our results rather support a role for APCs: when studying *in vitro* and *in vivo* T cell priming by DCs, we observed a trend toward an enhanced T cell activation by NOX2-deficient DCs *in vitro*, which was confirmed by an enhanced T cell activation *in vivo* in NOX2-deficient mice. However, we cannot exclude that NOX2 in other cell types might contribute to increase the T cell activation and altered IgG production. Indeed, fully functional NOX2 is expressed in B cells.

There is now increasing consensus that specific immunity, including humoral immune responses are enhanced in CGD patients. For most parts, these findings have also been documented in CGD mice. However, there are certain differences between the results reported in different studies that should be noted. Indeed, studies using the TLR4 ligand LPS as adjuvant, did not find increased T cell activation by phagocyte NADPH oxidase-deficient DCs (4, 31). Note that in our study, we have also observed differences between adjuvants, namely the use of alum vs. the dectin-1 ligand curdlan. Thus, different ways of DC activation yields distinct T and B cell responses, adding to the complexity of the specific immunity in CGD patients. At least two observations argue in favor of the *in vivo* relevance of our observations: (i) dectin-1 is increasingly recognized as a relevant activator of DCs (32) and (ii) altered IgG subtypes in CGD patients were identified in our study.

The enhanced T cell activation by NOX2-deficient DCs might be explained through at least four different mechanisms:

(i) Direct impact of ROS on T cells. Our study did not find direct impact of hydrogen peroxide on T cell activation (Figure 6A). Interestingly, other studies using adaptive transfer of athritogenic T cells derived from Ncf1-deficient rodents report that hydrogen peroxide attenuates the athritogenic properties of such T cells (30, 33). Thus, depending on the experimental set-up there might be a direct effect of hydrogen peroxide. Also, the primary product of NOX2 activation is the superoxide radical anion ${\text{O}}_{2}^{•-}$ and these experiments do not address the impact of NOX2-derived oxidants, other than H_2_O_2_.
(ii) Antigen processing in NOX2KO DCs. Some studies have described an altered antigen processing in NOX2KO DCs, resulting in an altered cross-presentation or in a different epitopic repertoire (19, 34–37). However, we have used the OVA_(323–339)_ peptide for our experiment on T cell activation, which does not require further peptidic cleavage to be presented through MHC class II molecules. Although, we cannot exclude that mechanism such as peptide transport and post-proteolytic modifications might be impacted by NOX2 deficiency, our results cannot be explained by a difference in proteases activity.
(iii) Alteration of surface proteins (in particular costimulatory molecules). We have investigated expression of costimulatory molecules by WT and NOX2-deficient DCs and did not observe any differences.
(iv) Altered release of soluble mediators (in particular cytokines): this mechanism is strongly supported by our data, as we find important differences in the release of two important T cell modulatory cytokines, namely IL1-β and IL-12, which polarize T cell toward a Th1 effector phenotype. Thus, in our hands, the release of soluble mediators from DCs is important for the understanding of the enhanced immune response in the CGD situation.

Another novel and unexpected result of both our *in vitro* and *in vivo* studies is the observation that the enhanced immune activation in NOX2-deficient mice concurred with an increased entry of T cells into the cell cycle, rather than an increased T cell proliferation rate. This observation is in line with our results suggesting that there is no cell autonomous effect of NOX2 in T cells, but rather that the initiation of T cell activation is controlled by NOX2 in DCs.

The human situation is more complex, due to first, the limitation of our knowledge on the IgG isotype switch, and second, the difficulty to perform clinical studies in CGD patients investigating the response to a specific antigen after immunization. Although the nomenclature is close between human and mice IgG subclass, their function differ in many aspects and direct comparison between high mouse IgG2c in mice and altered IgG1/IgG and IgG2/IgG ratio in human should be interpreted with caution. Nevertheless, our study has identified a remarkable, hitherto not described, pattern of IgG subtype distribution. The proportion of IgG2 was increased in adult CGD patients, while there was a relative decrease in IgG1. The increased levels of IgG2 were not observed at birth, but rather developed over the first 12 years of life. This suggests that there might be similarities between our experimental results obtained in NOX2-deficient mice and the condition of CGD children exposed to antigens. The age-dependent increase in IgG2 in human CGD patients might possibly reflect exposure to encapsulated bacteria (38). The increased IgG, and in particular IgG2, levels might provide compensatory mechanisms for the host defense against infections in CGD patients. However, such an increased propensity toward antibody generation is likely to come at a price. Indeed, as discussed in the Section "Introduction," CGD patients are prone to autoimmune diseases. In our serum samples from CGD patients, we found an increased proportion of positivity for the ANCA autoantibody (31%). In adult controls, only 6% (58 out of 924) serum samples were found positive using a similar IFI method (39). However, the results are largely variable among laboratories and methods. In addition, a further confounding factor is that ANCA formation is triggered by infection, and since CGD patients often encounter repeated infection, it would not be possible to exclude this as a cause of increased ANCA prevalence in these patients (40). ANA antibodies were positive in 3 out of 16 CGD patients (19%) while the percentage is 13.3% in control adults (41). The percentage of ANCA and ANA positivity is unknown in children, but autoantibody frequency usually increases with age and positivity is, therefore, likely to be decreased in children compared to adults. Altogether, our results confirm the increased prevalence of autoantibodies in CGD patients.

In summary, our results provide novel insights into the mechanisms underlying increased specific immune responses in CGD patients. Augmented cytokine production by NOX2-deficient DCs appears to be a crucial mechanism implicated in enhanced T cell activation and autoantibody production. Thus, targeting the overshooting release of T cell modulatory cytokines might be a promising approach for the treatment of hyperimmune disorders in CGD patients. It would, however, be beneficial to better understand how ROS dampen the release of cytokines in DCs. The identification of NOX2-derived ROS targets would open the path to novel immunomodulatory strategies at the interface of innate and specific immunity.

## Ethics Statement

Blood samples were obtained from the CGD patients with appropriate institutional informed consent. This study also includes peripheral blood samples taken from healthy donors obtained from the "Establishment Français du sang" at the Grenoble University Hospital, France after their informed consent. For animal subject, the protocol was approved by the office cantonal vétérinaire du Canton de Genève, Switzerland (authorization no. 23624).

## Author Conributions

JC conceived the study, designed and performed experiments, analyzed the data, and wrote the manuscript. CD helped in the design of the study and the analysis of data. MA edited the manuscript. PR-L performed the autoantibody titer in human. AG performed the measurement of serum immunoglobulin in human. MJS diagnosed and characterized the mutation of CGD patients. SH helped in the design of T cell experiment *in vivo* and *in vitro*. K-HK conceived the study, wrote and edited the manuscript. All authors read and approved the final manuscript.

## Conflict of Interest Statement

K-HK holds shares of Genkyotex SA, a company aiming at developing NOX inhibitors. The remaining authors declare that the research was conducted in the absence of any commercial or financial relationships that could be construed as a potential conflict of interest.

## Supplementary Material

Table S1 Demographic and blood biochemistry values for CGD patients and healthy controls. CGD patient samples are listed from 1–16, while healthy controls are assigned A–F. Values exceeding laboratory reference values are indicated in red shading. Reference values: IgG = 7–10 g/l; IgA = 0.9–2.3 g/l; IgM = 0.4–0.9 g/l; anti-MPO < 6 U; anti-PR3 < 5 U; and ANA < 80. Abbreviations: F, female; M, male; NA, not available; ND, not detected (below detection level); CGD, chronic granulomatous disease; A47^0^, p47*^phox^*-deficient autosomal recessive CGD; A67^0^, p67*^phox^*-deficient autosomal recessive CGD; A22^0^, p22*^phox^*-deficient autosomal recessive CGD; ANCA, anti-neutrophil cytoplasmic antibodies; ANA, anti-nuclear antibody.

Figure S1 Enhanced inflammation of draining lymph node: draining cervical and non-draining inguinal lymph nodes were removed 14 days post immunization, dissociated, and analyzed by flow cytometry: **(A)** absolute number of IgD^+^ B cells and **(B)** absolute number of CD4^+^ T cells.

Figure S2 Representative dot plot graphs showing CD69 expression in CD4-positive T cells after co-culture with different concentration of OVA_(323–339)_ peptide.

Figure S3 Representative histogram of carboxyfluorescein succinimidyl ester dilution at different concentration of OVA_323–339_ peptide after 3 days of co-culture with wild-type or NOX2KO BMDC.

Figure S4 NOX2 deficiency has no impact on the expression level of ICOSL **(A)**, PDL1 **(B)** and DC-SIGN **(C)** or the level of IL10 **(D)** present in the supernatant, after activation of BMDCs by increasing concentration of curdlan.
