# Supplementary material for: Altered Humoral Immune Responses and IgG Subtypes in NOX2-Deficient Mice and Patients: A Key Role for NOX2 in Antigen-Presenting Cells
Source: Front Immunol. 2018 Jul 11;9:1555. doi: 10.3389/fimmu.2018.01555 (PMC6050363; doi:10.3389/fimmu.2018.01555)
Supplement: Table S1 — Demographic and blood biochemistry values for CGD patients and healthy controls. CGD patient samples are listed from 1–16, while healthy controls are assigned A–F. Values exceeding laboratory reference values are indicated in red shading. Reference values: IgG = 7–10 g/l; IgA = 0.9–2.3 g/l; IgM = 0.4–0.9 g/l; anti-MPO < 6 U; anti-PR3 < 5 U; and ANA < 80. Abbreviations: F, female; M, male; NA, not available; ND, not detected (below detection level); CGD, chronic granulomatous disease; A470, p47phox-deficient autosomal recessive CGD; A670, p67phox-deficient autosomal recessive CGD; A220, p22phox-deficient autosomal recessive CGD; ANCA, anti-neutrophil cytoplasmic antibodies; ANA, anti-nuclear antibody. [file table_1.PDF]

Suppl. Table 1

Patient ID	CGD type	Sex	Age (year)	IgA (g/L)	IgG (g/L)	IgM (g/L)	IgG1 (g/L)	IgG2 (g/L)	IgG3 (g/L)	IgG4 (g/L)	ANCA	ANA
**1**	X-linked	M	1	<0,313	5,18	0,653	4,27	0,82	0,126	0,0115	NEG	NEG
**2**	X-linked	M	1,5	1,11	12,8	2,39	10,2	1,79	0,518	0,0561	POS	NEG
**3**	X-linked	M	1,5	0,845	8,01	1,64	6,37	1,24	0,205	0,114	POS	NEG
**4**	X-linked	M	1,5	1,18	10,4	1,29	8,51	0,481	0,207	0,0191	NEG	NEG
**5**	A22^0	F	1,5	<0,313	12,6	0,498	10,6	1,39	0,434	0,177	POS	NEG
**6**	X-linked	M	2	2,33	13	1,12	11,7	1,46	0,226	0,0346	NEG	NEG
**7**	A47^0	M	2	2,56	9,63	0,796	7,65	1,7	0,268	0,0234	NEG	NEG
**8**	A67^0	F	3	1,81	12,3	0,957	8,48	2,12	0,184	1,09	POS	POS
9	A22^0	F	*3,5*	*0,846*	*9,63*	*0,446*	*6,19*	*3,45*	*0,149*	*0,596*	NEG	NEG
10	A22^0	F	*4*	*3,87*	*9,32*	*0,633*	*4,51*	*4,95*	*0,0819*	*0,254*	NEG	NEG
**11**	A22^0	F	12	0,852	5,82	0,403	3,64	2,01	0,13	0,0386	NEG	POS
**12**	A47^0	M	20	3,06	6,29	0,591	4,37	1,89	0,164	0,135	POS	POS
**13**	X-linked	M	27	4,13	13,4	0,879	6,85	6,09	0,269	0,216	POS	NEG
**14**	A47^0	M	27	1,95	18,9	1,03	11	9,47	0,506	0,78	NEG	NEG
**15**	A47^0	M	29	6,98	12,3	1,31	6,38	6,47	0,266	0,613	NEG	NEG
**16**	X-linked	M	29	2,75	10,2	0,728	5,6	4,76	0,0817	0,457	NEG	NEG
**Healthy controls**												
**A**		F	20	0,624	9,48	0,82	6,76	2,78	0,181	2,88		
**B**		F	28	1,14	6,55	0,966	4,59	1,16	0,279	0,0906		
**C**		F	33	4,14	7,42	1,81	4,93	2,62	0,339	0,0328		
**D**		F	39	1,63	9,15	0,569	6,72	2,43	0,197	0,752		
**E**		F	52	1,73	8,55	0,672	5,86	2,22	0,173	0,265		
**F**		NA	adult	2,66	8,93	0,754	5,22	2,9	0,945	0,399		
