# Supplementary figures and images for: Altered Humoral Immune Responses and IgG Subtypes in NOX2-Deficient Mice and Patients: A Key Role for NOX2 in Antigen-Presenting Cells
Source: Front Immunol. 2018 Jul 11;9:1555. doi: 10.3389/fimmu.2018.01555 (PMC6050363; doi:10.3389/fimmu.2018.01555)

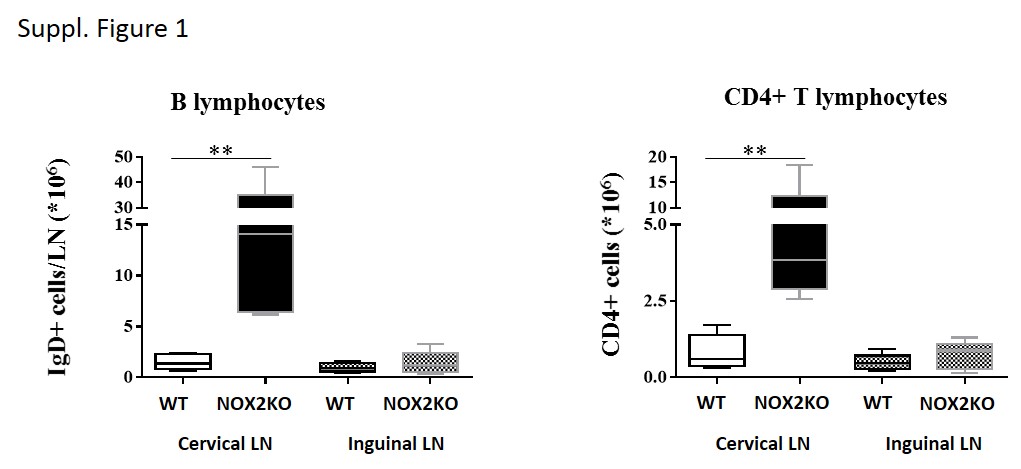

Supplement: Figure S1 — Enhanced inflammation of draining lymph node: draining cervical and non-draining inguinal lymph nodes were removed 14 days post immunization, dissociated, and analyzed by flow cytometry: (A) absolute number of IgD+ B cells and (B) absolute number of CD4+ T cells. [file image_1.jpg]

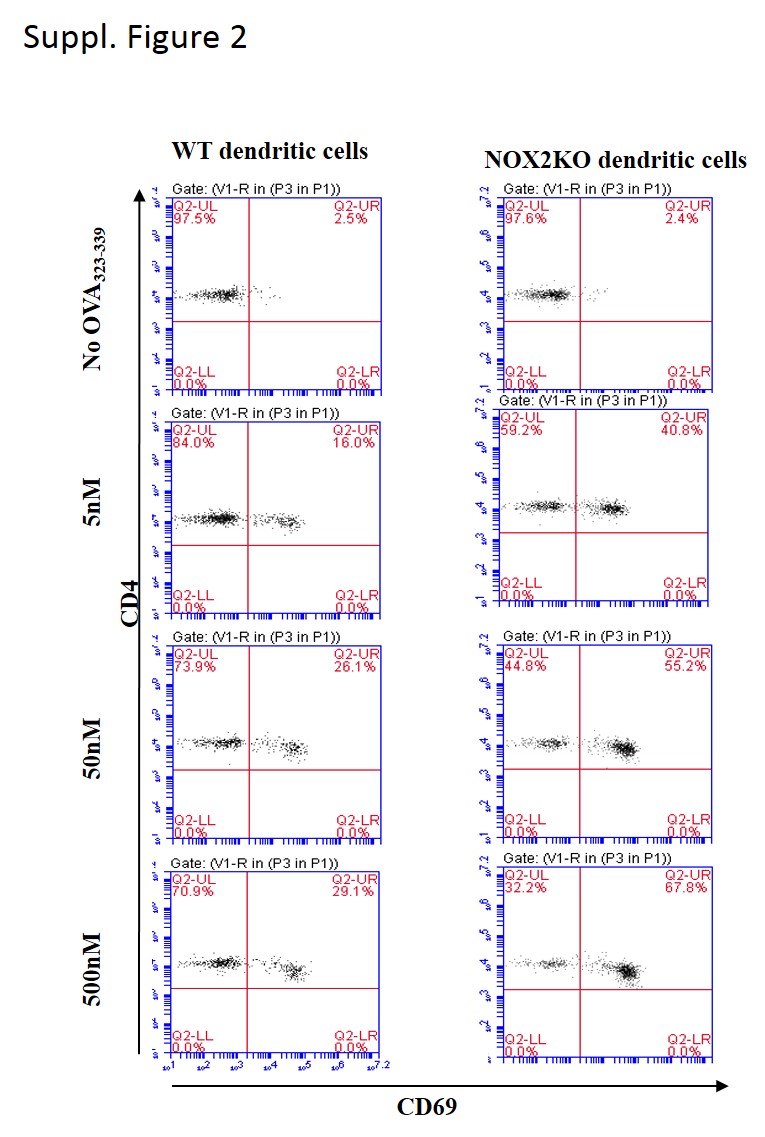

Supplement: Figure S2 — Representative dot plot graphs showing CD69 expression in CD4-positive T cells after co-culture with different concentration of OVA(323–339) peptide. [file image_2.jpg]

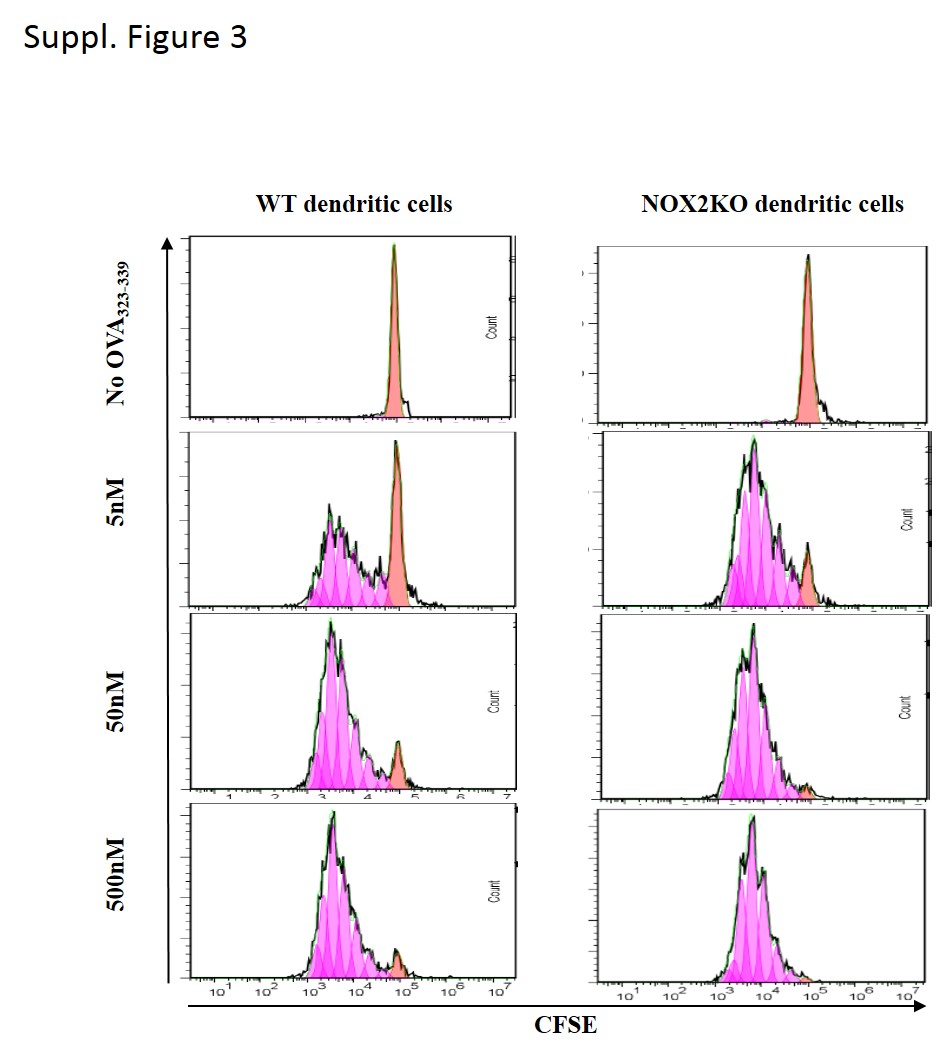

Supplement: Figure S3 — Representative histogram of carboxyfluorescein succinimidyl ester dilution at different concentration of OVA323–339 peptide after 3 days of co-culture with wild-type or NOX2KO BMDC. [file image_3.jpg]

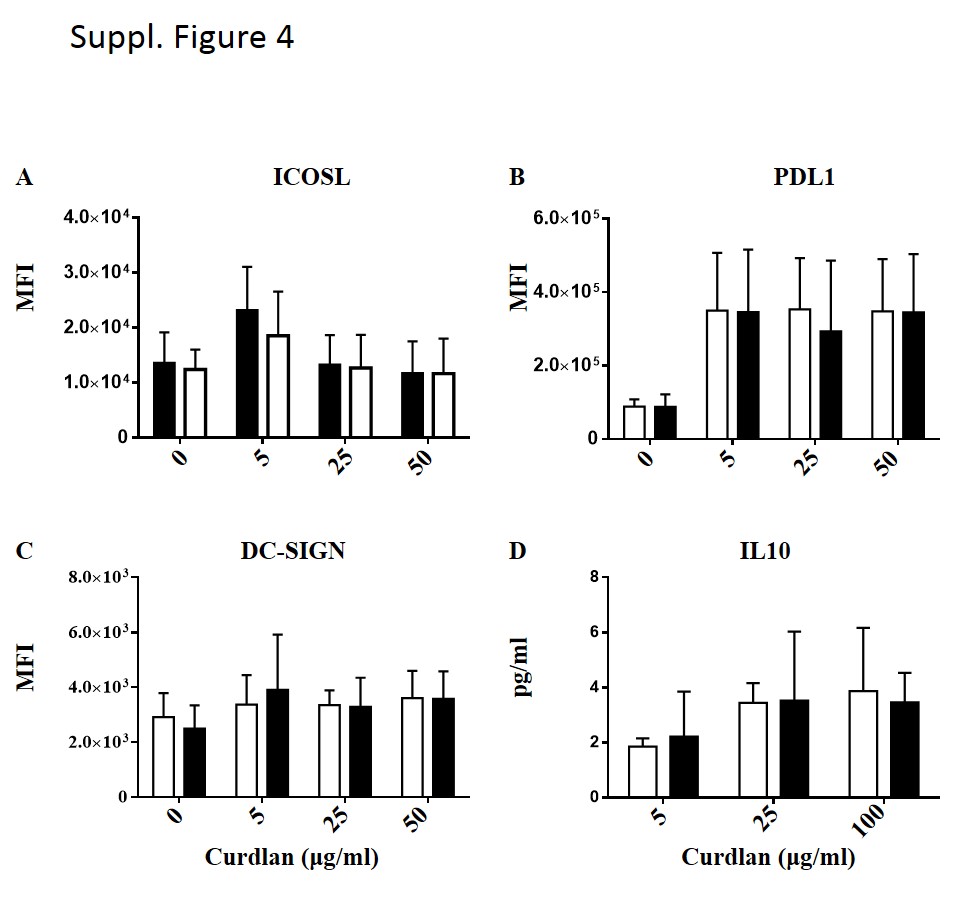

Supplement: Figure S4 — NOX2 deficiency has no impact on the expression level of ICOSL (A), PDL1 (B) and DC-SIGN (C) or the level of IL10 (D) present in the supernatant, after activation of BMDCs by increasing concentration of curdlan. [file image_4.jpg]
